# CD161 Defines a Functionally Distinct Subset of Pro-Inflammatory Natural Killer Cells

**DOI:** 10.3389/fimmu.2018.00486

**Published:** 2018-04-09

**Authors:** Ayako Kurioka, Cormac Cosgrove, Yannick Simoni, Bonnie van Wilgenburg, Alessandra Geremia, Sophia Björkander, Eva Sverremark-Ekström, Christine Thurnheer, Huldrych F. Günthard, Nina Khanna, The Swiss HIV Cohort Study, V Aubert, Oxford IBD Cohort Investigators, CV Arancibia-Cárcamo, Lucy Jane Walker, Carolina V. Arancibia-Cárcamo, Evan W. Newell, Christian B. Willberg, Paul Klenerman

**Affiliations:** ^1^The Peter Medawar Building for Pathogen Research, University of Oxford, Oxford, United Kingdom; ^2^Ragon Institute of Massachusetts General Hospital, Harvard University, Massachusetts Institute of Technology, Cambridge, MA, United States; ^3^Agency for Science, Technology and Research (A*STAR), Singapore Immunology Network (SIgN), Singapore, Singapore; ^4^Translational Gastroenterology Unit, Experimental Medicine Division, University of Oxford, John Radcliffe Hospital, Oxford, United Kingdom; ^5^Department of Molecular Biosciences, The Wenner-Gren Institute, Stockholm University, Stockholm, Sweden; ^6^Division of Infectious Diseases, University Hospital Berne, University of Berne, Berne, Switzerland; ^7^Division of Infectious Diseases and Hospital Epidemiology, University Hospital Zurich, University of Zurich, Zurich, Switzerland; ^8^Institute of Medical Virology, University of Zurich, Zurich, Switzerland; ^9^Division of Infectious Diseases, University Hospital Basel, Basel, Switzerland; ^10^Institute of Cellular Medicine, Newcastle University, Newcastle upon Tyne, United Kingdom; ^11^NIHR Biomedical Research Centre, University of Oxford, Oxford, United Kingdom

**Keywords:** natural killer cells, CD161, pro-inflammatory cytokines, cytomegalovirus, human immunodeficiency virus, inflammatory bowel diseases

## Abstract

CD161 is a C-type lectin-like receptor expressed on the majority of natural killer (NK) cells; however, the significance of CD161 expression on NK cells has not been comprehensively investigated. Recently, we found that CD161 expression identifies a transcriptional and innate functional phenotype that is shared across various T cell populations. Using mass cytometry and microarray experiments, we demonstrate that this functional phenotype extends to NK cells. CD161 marks NK cells that have retained the ability to respond to innate cytokines during their differentiation, and is lost upon cytomegalovirus-induced maturation in both healthy and human immunodeficiency virus (HIV)-infected patients. These pro-inflammatory NK cells are present in the inflamed lamina propria where they are enriched for integrin CD103 expression. Thus, CD161 expression identifies NK cells that may contribute to inflammatory disease pathogenesis and correlates with an innate responsiveness to cytokines in both T and NK cells.

## Introduction

Natural killer (NK) cells are the most classical population of innate lymphoid cells, expressing a heterogeneous repertoire of germline-encoded receptors that allows them to distinguish infected or stressed cells from healthy cells (1). CD161 is one of the earliest markers expressed during NK cell maturation from CD34^+^ hematopoietic stem cell precursors (2). Expression of CD161 correlates with the cytotoxic function of CD16^+^ NK cells (3), and ligation of CD161 with its ligand LLT1 inhibits NK cell cytotoxicity and cytokine secretion (4, 5).

CD161 is expressed early in NK cell development, where it may facilitate cross-talk of NK cell precursors with cells within the bone marrow, and is involved in CXCL8 release (6–8). Within the periphery, cross-linking of CD161 leads to an increase in IFNγ expression and inhibition of NK cell cytotoxicity (4, 5, 9, 10).

There have been various reports of modulated expression of CD161 on NK cells during viral infections. For instance, reduced CD161 expression in acute hepatitis C virus (HCV) infection predicted viral clearance (11), and correlated with increased liver inflammation in chronic HCV infection (12). Patients with chronic human immunodeficiency virus (HIV) infection have depleted CD161^+^ NK cells compared to healthy donors (13, 14). Recently, a population of NK cells with memory-like properties has been described in the context of cytomegalovirus (CMV) (15, 16), referred to as “adaptive” NK cells (17). This subset expands in recipients of solid organ (18) or umbilical cord blood transplantation (19) during primary CMV infection or reactivation. These cells are commonly NKG2C^+^ and CD57^++^ (18, 20, 21), and CD161 expression on these cells has been shown to be reduced (17, 20). Overall, despite reports on the expression of CD161 in different viral infections, our understanding of its significance—i.e., the function of NK cells based on CD161 expression—is incomplete.

Pro-inflammatory cytokines strongly influence the activation of NK cells against viral infections, promoting their cytotoxicity, expansion, and production of immunomodulatory cytokines. IL-12 and IL-18, for example, induce IFNγ production from NK cells which is critical for NK cell-mediated protection in murine CMV (MCMV)-infected livers (22) and is required for the primary expansion of memory NK cell responses against MCMV infection (23, 24). Recently, we have demonstrated that CD161-expressing T cells share a transcriptional signature, regardless of T cell receptor (TCR) expression (25), which is associated with an increased responsiveness to IL-12 and IL-18. Whether this transcriptional signature and functional correlation with CD161 holds true within NK cells is unknown.

Using flow and mass cytometry, we show in this study that CD161 expression marks pro-inflammatory NK cells with high cytokine responsiveness, independent of CMV status or CD57 expression. These cells share a transcriptional signature with CD161^+^CD8^+^T cells and express the integrin CD103 in the inflamed gut of inflammatory bowel disease (IBD) patients. Furthermore, we find that CD161 expression is lost from NK cells in a CMV-dependent manner from chronic HIV patients from the Swiss HIV cohort study (SHCS), which partially recovers with anti-retroviral therapy (ART). Overall, these data define the functional correlates of CD161 expression, which can be modulated by infection and inflammation.

## Materials and Methods

### Cells

Peripheral blood mononuclear cells (PBMCs) were obtained from adults (whole blood leukocyte cones, NHS Blood and Transplant or laboratory volunteers, numbers varied for individual experiments and are indicated in each figure), 24-month-old donors (prospective birth cohort (26), *n* = 28) and umbilical cord blood samples (Stem Cell Services, NHS Blood and Transplant, *n* = 5). Intestinal lamina propria cells were isolated following EDTA washes by Percoll gradient centrifugation (*n* = 7; Table S1 in Supplementary Material). Where indicated, PBMCs were obtained from a cohort of treatment-naïve patients from the SHCS(*n* = 27), sampled prior to, and 1 year and 2 years into ART (Table S2 in Supplementary Material).

Adult and cord blood samples were collected after ethical approval by the Central Office for Research Ethics Committees (COREC, local research ethics committee Oxford), reference number COREC 04.OXA.010. The NHS Research Ethics System provided ethical approval for the Oxford IBD Cohort study (reference numbers 09/H0606/5 for IBD patients and 11/YH/0020 and 16/YH/0247 for controls). All patients from the studies above provided their informed written consent. The collection of blood samples for the 24-month-old study cohort was approved by the Human Ethics Committee at Huddinge University Hospital, Stockholm, reference code 75/97, 331/02, and the parents provided their informed verbal consent. No written documentation of the participants informed approval was required, which was agreed to by the Human Ethics Committee and was according to the regulations at the time of the initiation of the study.

The SHCS was approved by the local ethical committees of the participating centers: Kantonale Ethikkommission Zürich (KEK-ZH-NR: EK-793); Ethikkommission beider Basel (“Die Ethikkommission beider Basel hat die Dokumente zur Studie zustimmend zur Kenntnis genommen und genehmigt.”); Kantonale Ethikkommission Bern (21/88); Comité departmental d’éthique des specialités médicales es de médecine communautarie et de premier recours, Hôpitaux Universitaires de Genève (01–142); Commission cantonale d’éthique de la recherche sur l’être humain, Canton de Vaud (131/01); Comitato etico cantonale, Repubblica e Cantone Ticino (CE 813); Ethikkommission des Kantons St. Gallen (EKSG 12/003), and written informed consent was obtained from all participants.

### Flow Cytometry

Antibodies used were as follows: CD56 BV421, CD3 PE-Cy7 or APC-Cy7, CD14 APC-Cy7, CD19 APC-Cy7, CD57 Pacific Blue or FITC, CD62L PE-Cy7, CD107a PE-Cy7, CD244 PE-Cy5.5, Perforin Pacific blue, CD160 Alexa Fluor 647 (Biolegend), CD16 eFluor450 or FITC, CD69 FITC, CD94 FITC, CD8a PerCP-Cy5.5 (eBioscience), CD161 PE or APC, CD56 APC, IFNγ FITC, NKp30 APC, NKp46 APC, NKp80 APC (Miltenyi Biotec), CD3 Pacific Orange, CD4 Qdot 605, Granzyme B APC (Invitrogen), NKG2C Alexa Fluor 488 or PE, NKG2D PE, PLZF APC, Granzyme A FITC (R&D Systems), CD56 FITC or PE-Cy7, CD85j FITC, IFNγ Alexa Fluor 700, Ki67 FITC (BD Biosciences), Granzyme K FITC (Immunotools), NKG2A PE, NKp44 PE, CD158e1/e2 PE (Beckman Coulter), Phosphatidylserine Alexa Fluor 488 (Merck Milipore). The viability dye Live/Dead fixable Near-IR (Invitrogen) was used in all experiments. Anti-KLRG1 FITC was kindly provided by H. Pircher. Data were acquired on LSRII (BD Biosciences) and analyzed using FlowJo (Treestar, Inc.).

### *In Vitro* Assays

Peripheral blood mononuclear cells were cultured for 20 h with 50 ng/ml IL-12 and IL-18 (Miltenyi Biotec) and Brefeldin A (eBioscience) added for the last 4 h. To determine CMV status in healthy donors of unknown CMV status, PBMCs were cultured with CMV lysate for 16 h at 5 µg/ml (Virusys Corporation), with Brefeldin A (eBioscience) added after 1 h of stimulation, followed by analysis of IFNγ secretion. CMV+ donors were defined as individuals with IFNγ^+^CD4^+^T cells in response to CMV-lysate above background (average % IFNγ^+^CD4^+^ T cells = 0.02% in CMV− vs. 2.42% in CMV^+^ donors). In addition, healthy laboratory donors with known CMV seropositivity were included as CMV^+^ donors.

Alternatively, PBMCs or purified CD161^+^ or CD161^−^ NK cells (sorted on a MoFlo, Beckman Coulter) were labeled with 5 µM CellTrace Violet (CTV; Invitrogen) according to the manufacturer’s protocol and cultured with: IL-2 (100 IU/ml; Roche Diagnostics), IL-15 (25 ng/ml; Miltenyi Biotec), Phytohemagglutinin (PHA; 2 µg/ml, Sigma Aldrich), IL-18 or IL-12 (both 50 ng/ml; Miltenyi Biotec), or combinations of stimuli for 6 days. Where indicated cells were stained with phosphatidylserine AlexaFluor488 (Millipore) following proliferation. Alternatively, CTV-labeled PBMCs were cultured on flat bottom ELISA plates (Greiner Bio-One Limited) coated with purified anti-CD16 (BD Biosciences), anti-NKG2C (R&D Systems), or isotype control (BD Biosciences).

### Microarray Analysis

CD161^+^CD161^+^ or CD161^−^ NK cells (singlet, alive, CD14^−^CD19^−^CD3^−^CD56^+^) were sorted using a MoFlo MLS cell sorter (Beckman Coulter) from four donors. Purity was >96%. Three out of four donors were CMV seronegative, while the seropositivity of the remaining donor is unknown. Cell pellets were snap frozen and sent to Miltenyi Biotec Genomic Services (Bergisch Gladbach) for RNA extraction and hybridization to Agilent Whole Human Genome Oligo Microarray. Raw microarray image files were processed using Agilent feature extraction, and differential gene expression was analyzed using the Rosetta Resolver gene expression data analysis system (Rosetta Biosoftware). Hierarchical clustering of differentially regulated genes (>2-fold, *p* < 0.01) was completed using the heatmap function in GENE-E (Broad Institute). The NCBI Gene Expression Omnibus accession number for the microarray data reported in this paper is GSE98702. The NK cell data were compared to genes significantly upregulated (>2-fold, *p* < 0.05) in a previously published dataset of CD161^intermediate(int)^ CD8^+^ T cells compared to the CD161^−^CD8^+^T cells (25, 27, 28) using gene set enrichment analysis (GSEA) v2.1.0 (29).

### Mass Cytometry

Frozen PBMCs were thawed and washed in RPMI 1640 with 10% fetal calf serum (FCS), supplemented with penicillin/streptomycin and l-glutamine (R10) with DNAse (all from Sigma Aldrich). PBMCs were depleted of CD4^+^T cells, monocytes, and B cells, using anti-Mouse IgG Microbeads (Miltenyi Biotec) following staining with anti-CD4, or anti-CD33^+^anti-CD13, or anti-CD20 antibodies, respectively. Cells were cultured in RPMI 10% FCS for 20 h with IL-12 and IL-18 (both 50 ng/ml, Miltenyi Biotec), and 1X Brefeldin A (eBioscience) was added at 3 µg/ml for the last 4 h. Cells were first stained with the viability marker Cisplatin (Sigma Aldrich) at 5 µM in PBS for 5 min on ice, then with metal-conjugated surface antibodies and fluorochrome-conjugated antibodies, in RPMI+10% FCS at 37°C for 15 min. After washing, cells were stained in 1% bovine serum albumin/PBS buffer with the remaining surface antibodies, including secondary antibodies against the fluorescent tags, at 4°C for 15 min. Cells were then fixed in Fixation/Permeabilization buffer (eBioscience) for 30 min at 4°C. Following washing in Permeabilization buffer (eBioscience), cells were incubated with intracellular antibodies during 30 min at 4°C in Permeabilization buffer. A unique dual combination of metal barcodes was added to each sample for barcoding as previously described (30, 31) and cells were DNA labeled with 250 nM iridium interchelator in 2% PFA (DVS Sciences). Cells were fixed in 2% PFA overnight at 4°C. Cells were washed twice before dilution to 5 × 10^5^ cells/ml in distilled water before being acquired on a CyTOF^TM^-2 instrument (Fluidigm). Gating strategy is shown in Figure S1 in Supplementary Material. Details of antibodies used are shown in Table S3 in Supplementary Material.

t-Distributed stochastic neighbor embedding (t-SNE) analysis was either performed in Cytobank (www.cytobank.org; Cytobank Inc.) or in R. Biaxial gating was performed on FlowJo Version 10 software (Treestar).

Fetal calf serum files were imported into R *via* the read.FCS function in the flowCore package, as described previously (31). t-SNE analysis was performed using custom R scripts using R packages that perform the Barnes-Hut implementation of t-SNE. Cells from each cluster identified by t-SNE were grouped and the median intensity values for each cluster for every marker was calculated for the generation of heatmaps. For Cytobank analysis, live, CD45^+^CD14^−^CD19^−^FcεR1^−^CD123^−^CD11c^−^ cells were gated (excluding monocytes, myeloid and plasmacytoid DC, mast/basophils, and B cells), and t-SNE analysis was performed based on the remaining parameters with proportional sampling, so that the algorithm samples from gated populations preserving their relative abundance. For further NK cell analysis, CD3^−^CD5^−^CD56^+^ cells were gated within these cells using Cytobank, exported, and reanalyzed in Cytobank.

### Statistical Analysis

For multiple group comparisons, one-way ANOVA or two-way ANOVA tests with Dunnett’s, Tukey’s, or Bonferroni’s multiple comparisons tests were applied. For single comparisons of matched groups, the paired Student’s *t*-test was performed. All figures present data as means ± SEM, ****p < 0.0001, ***p < 0.001, **p < 0.01, *p < 0.05, and ns = non-significant. Analyses were performed using Prism software (GraphPad).

## Results

### CD161 Expression Defines Two Distinct Subsets of NK Cells

**Figure 1:**
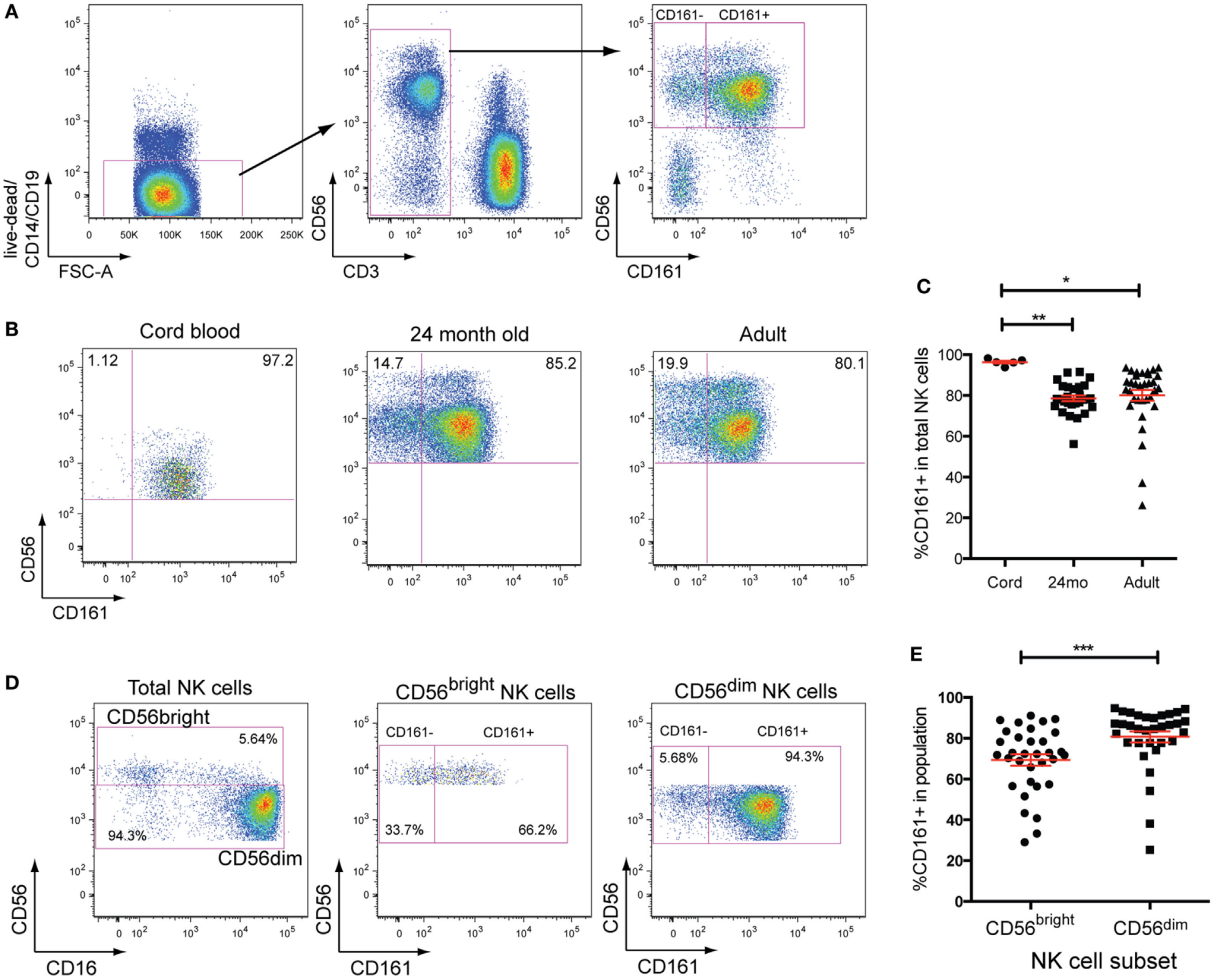
CD161 expression defines two distinct subsets of natural killer (NK) cells. **(A)** Gating strategy used to identify CD161^+^ and CD161^−^ NK cells. **(B)** Representative staining showing the expression of CD161 in umbilical cord blood, 24-month olds (24mo), and adult individuals on total NK cells (CD3^−^CD56^+^ cells). **(C)** Frequencies of CD161^+^NK cells in cord blood (*n* = 5), 24-month olds (*n* = 28), and adult individuals (*n* = 33). **(D)** Representative staining showing the expression of CD161 on CD56bright and CD56^dim^NK cells in adult peripheral blood. **(E)** Frequencies of CD161^+^ cells within CD56^bright^ and CD56^dim^NK cells (*n* = 33).

Natural killer cells were defined as CD19^−^CD14^−^CD3^−^CD56^+^ cells in this study. CD161 expression divides peripheral blood NK cells into two distinct populations in healthy adult donors (Figure 1A). Analysis of CD161 expression within cord blood samples, however, showed that NK cells were almost all CD161^+^ (Figures 1B,C), in line with previous reports (2). The frequency of CD161^+^NK cells did not differ between 24-month-old and adult donors (Figure 1C), suggesting that the CD161^−^ NK cells expand within the first 2 years of life. Dissection of NK cells into the CD56^bright^ and CD56^dim^ NK cell populations (Figures 1D,E) showed that there is a substantial overlap between the populations, although there was a slight but significant enrichment of CD161^+^ cells within the CD56^dim^ subset.

### CD161^+^NK Cells Share High Responsiveness to IL-12 and IL-18 with CD161-Expressing T Cells

**Figure 2:**
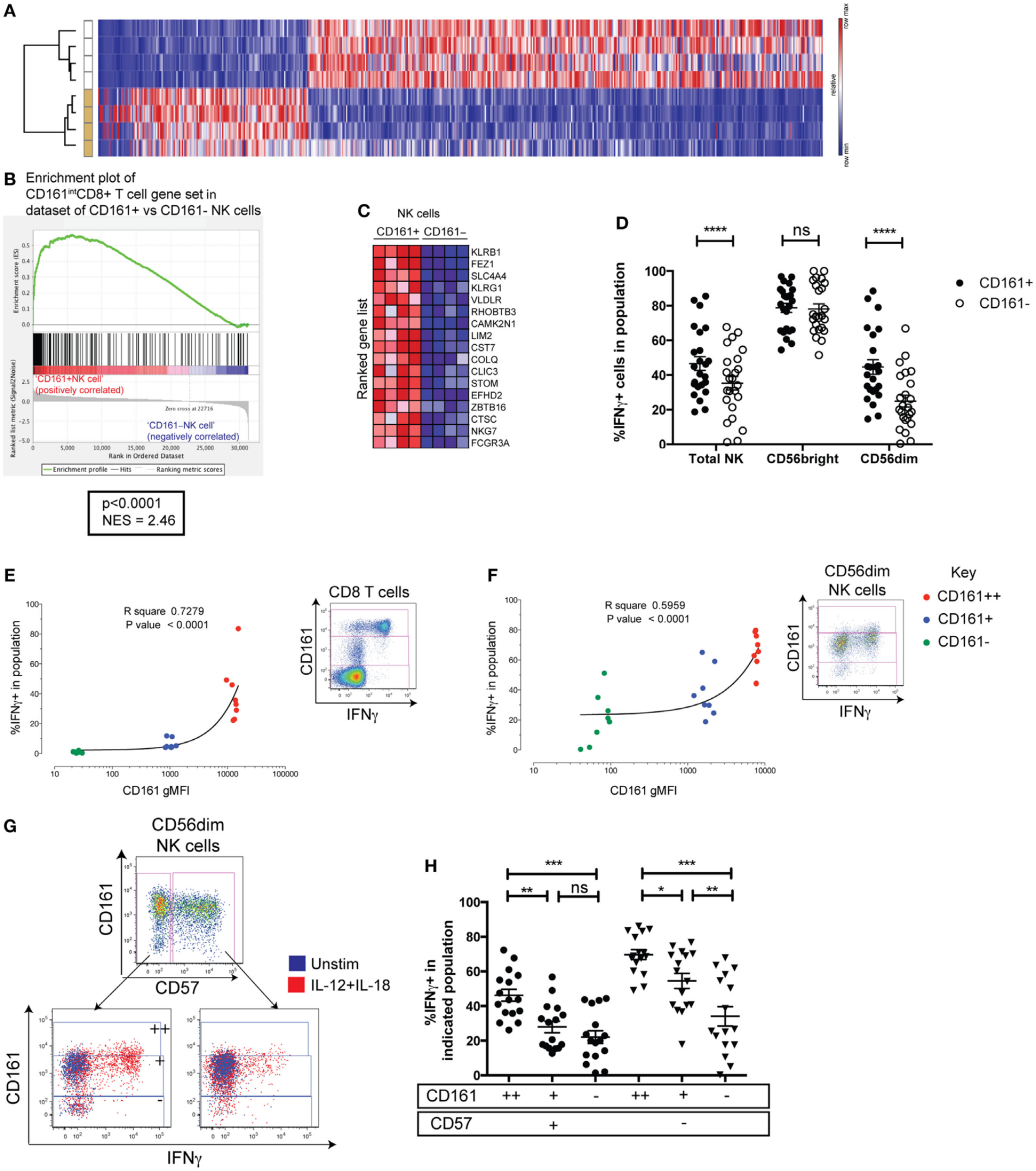
CD161^+^NK cells share high responsiveness to IL-12+IL-18 with CD161-expressing T cells. **(A)** Heatmap illustrating significantly differentially expressed transcripts between CD161^+^ and CD161^−^ NK cells in four donors. Subsets clustered by one minus Pearson correlation by GENE-E. **(B)** Gene set enrichment analysis of CD161^+^ vs. CD161^−^ NK cells, showing a significant enrichment of genes upregulated in CD161^+(intermediate)^ (CD161^int^) CD8^+^T cells compared to CD161^−^CD8^+^T cells (red = positively correlated, blue = negatively correlated) within CD161^+^ NK cells compared to CD161^−^ NK cells. **(C)** Heatmap shows top 17 genes that are highly (red) or lowly (blue) expressed in either CD161^+^ or CD161^−^ NK cells. NES = normalized enrichment score. **(D)** IFNγ production by CD161^+^ and CD161^−^ NK cells in response to overnight IL-12+IL-18 stimulation. *N* = 24. **(E,F)** gMFI of CD161 for CD161^bright (++)^ (red), CD161^int(+)^ (blue), and CD161^−^ (green) subsets in CD8^+^T cells **(E)** or CD56^dim^NK cells **(F)**, correlated with IFNγ expression in response to IL-12+IL-18 (*n* = 9). Representative staining showing IFNγ expression on indicated cells from the same donor, and gating of CD161^bright (++)^, CD161^int(+)^, and CD161^−^ subsets based on CD8^+^T cells, are shown. **(G)** Representative staining showing IFNγ production in response to IL-12+IL-18 from CD57^+^ and CD57^−^ cells within the CD56^dim^ natural killer (NK) cell population (left). CD161^bright (++)^, CD161^int(+)^, and CD161^−^ subsets were gated within CD57^+^ and CD57^−^ NK cells as indicated. **(H)** Frequency of IFNγ+ cells in each population was graphed.

To further explore the CD161^+^NK cell phenotype, mRNA microarray analysis was performed on FACS-sorted CD161^+^ and CD161^−^ NK cell populations. Based on a cutoff of a twofold difference in transcript abundance and a *p*-value of less than 0.01, 642 genes were identified as differentially regulated between the two subsets (Figure 2A; genes listed in Table S4 in Supplementary Material). Differential expression of some markers between the two subsets was confirmed at the protein level, and further phenotyping of receptors (Figure S2 in Supplementary Material) and cytotoxic molecules (data not shown) was performed, but none of the markers investigated were exclusive to CD161^+^ or CD161^−^ NK cells. Moreover, there was no difference in surface CD107a expression after co-culture with K562 target cells between these populations (Figure S3H in Supplementary Material).

Recently, we have found that CD161 expression on T cells identifies cells that share a core transcriptional signature, regardless of their lineage (25). Therefore, we investigated whether genes upregulated within CD161^+^NK cells were also shared with CD161-expressing T cells using GSEA (29). We tested whether the set of genes upregulated in peripheral CD8^+^T cells expressing CD161 at an intermediate level (CD161^int^) (28) compared to their CD161^−^ counterparts was enriched significantly in either the CD161^+^ or CD161^−^ NK cell population. There was a significant enrichment of genes upregulated in CD161^int^ (28) in comparison to CD161^−^CD8^+^T cells within CD161^+^NK cells (normalized enrichment score = 2.46, *p* < 0.0001; Figures 2B,C). These genes included *ZBTB16*, which encodes the transcription factor PLZF that directs the innate-like effector functions of T cells (32), and suggests that CD161^+^ NK cells and CD161^int^ CD8^+^ T cells share a transcriptional signature. This was unique to CD161^int^ CD8^+^ T cells, as there was no significant enrichment of genes upregulated in CD161^bright^^(++)^CD8^+^ T cells, CD161^+^CD4^+^ T cells, or CD161^+^ γδ T cells compared to their CD161^−^ counterparts within CD161^+^ NK cells (data not shown).

We have previously found that CD161 expression on T cells identifies cells with the ability to respond to IL-12 and IL-18 in a TCR-independent, “innate” manner (25). To investigate whether this functional correlate with CD161 expression extended beyond T cells, IFNγ production in response to IL-12+IL-18 was assayed in CD161^+^ and CD161^−^ NK cells. This showed that although the majority of CD56^bright^ NK cells secreted IFNγ regardless of CD161 expression, a consistently higher frequency of CD161-expressing CD56^dim^ NK cells produced IFNγ compared to their CD161^−^ NK cell counterparts (*p* < 0.0001) (Figure 2D). CD56 and CD161 expression was stable in most individuals tested after overnight stimulation, although there was a marginal increase in CD161 expression (Figures S3A,B in Supplementary Material). Furthermore, we have shown previously that the frequency of IFNγ-expressing cells correlates with the level of CD161 expressed when gating on CD161^bright (++)^, CD161^int(+)^, and CD161^−^ CD8^+^ T cells (Figure 2E). This correlation can also be seen in other T cells such as CD4^+^ T cells and γδ T cells (25). We found that gating on corresponding CD161-expressing populations in NK cells, IFNγ induction in response to IL-12 and IL-18 also strongly correlated with CD161 expression in CD56^dim^NK cells (Figure 2F) and the total CD56^+^ populations (Figure S3D in Supplementary Material), but not the CD56bright (Figure S3F in Supplementary Material). These results suggest that CD161 expression marks CD56^dim^ cells with an increased ability to respond to IL-12 and IL-18, which is shared with CD161-expressing T cells. Presumably, this is due to the correlation between CD161 and IL-18 receptor expression seen in the CD56dim (Figures S3E in Supplementary Material) and CD56bright populations (Figures S3G in Supplementary Material), although IL-18 receptor expression is already high on all CD56bright cells—explaining the lack of correlation between CD56 and IFNγ in this population. Of note, CD57 expression has been shown to be a major determinant in the responsiveness of CD56^dim^ NK cells to IL-12 and IL-18 (33, 34) and marks CMV-induced terminally differentiated NK cells that have a reduced capacity to respond to IL-12 and IL-18 (17). However, gating on CD161^bright (++)^, CD161^int(+)^, and CD161^−^ subsets within CD56^dim^ NK cells showed that the frequency of IFNγ^+^ cells clearly increased with increasing CD161 expression within both CD57^+^ and CD57^−^ CD56^dim^ NK cells, indicating that CD161 expression correlates with IL-12 and IL-18 responsiveness, independently of CD57 (Figures 2G,H).

### CD161^+^ NK Cells Are Highly Proliferative Compared to CD161^−^ NK Cells

**Figure 3:**
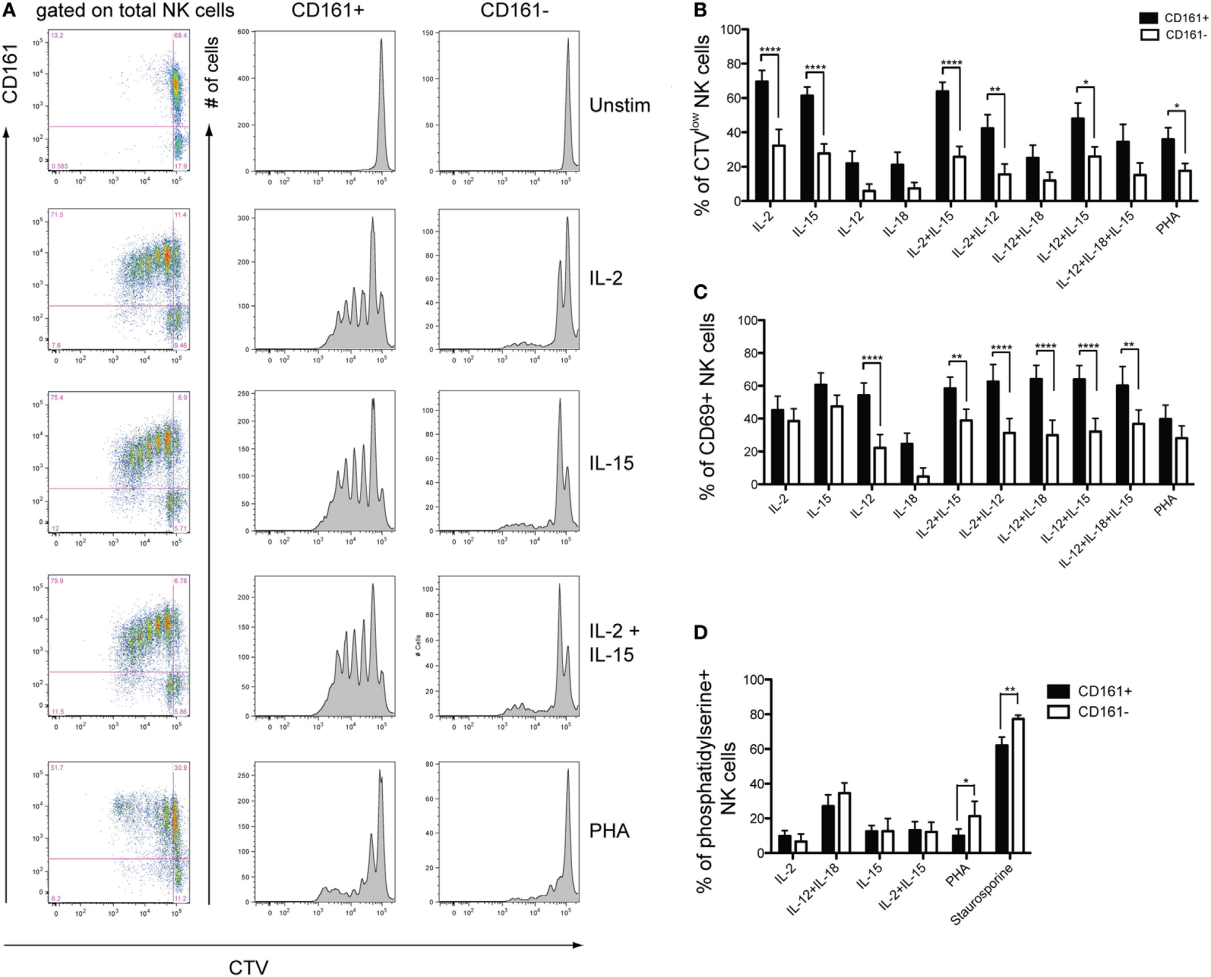

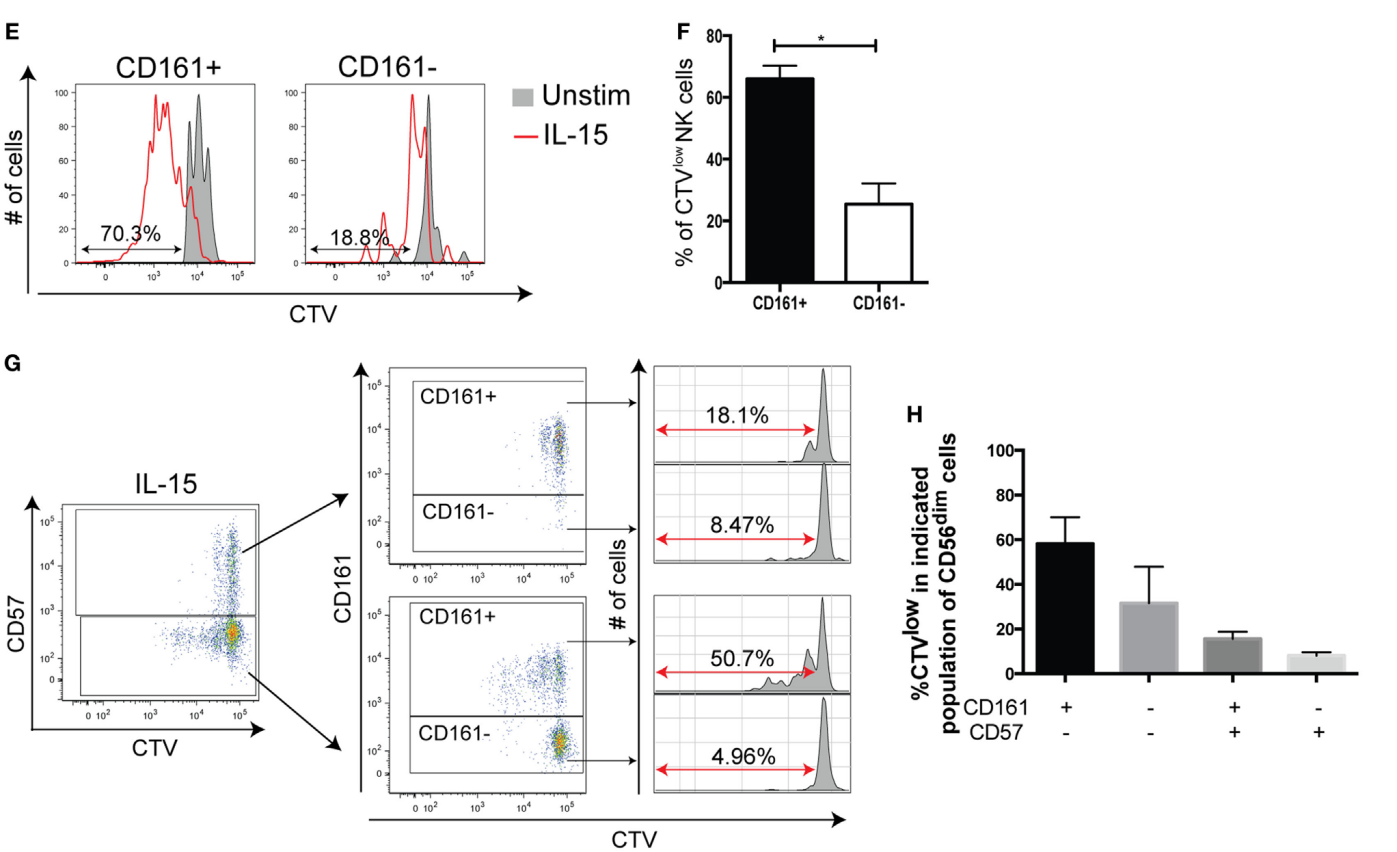
CD161^+^ natural killer (NK) cells have a greater proliferative capacity than CD161^−^ NK cells. Peripheral blood mononuclear cells from healthy donors were CTV-labeled and cultured for 5 days with the indicated stimuli. **(A)** Representative staining of CellTrace Violet (CTV) for CD161^+^ and CD161^−^ NK cells from one healthy donor. **(B,C)** Frequency of CD161^+^ and CD161^−^ NK cells either **(B)** diluting CTV, or **(C)** expressing CD69, after simulation with the indicated combinations of cytokines and PHA (*n* = 14). Graphs show frequencies of CTV^low^ and CD69^+^ cells following background subtraction. **(D)** Frequency of CD161^+^ and CD161^−^ NK cells expressing phosphatidylserine after 5 days culture with the indicated stimuli (*n* = 4). **(E,F)** CTV-labeled FACS-sorted CD161^+^ and CD161^−^ NK cells were stimulated with IL-15 for 5 days or left unstimulated, and the frequency of cells diluting CTV (CTV^low^) was analyzed (*n* = 3). **(E)** Representative staining and **(F)** frequency data are shown. **(G)** Representative staining for CTV on CD57^+^ and CD57^−^ NK cells, according to CD161 expression. **(H)** Frequency of cells diluting CTV on indicated CD56^dim^ NK cell subsets stimulated with IL-15, according to CD161 and CD57 expression (*n* = 3). CD56^bright^NK cells were excluded as they do not express CD57.

As CD161^+^ NK cells had a significantly higher expression of the proliferative marker Ki67 at the gene level (Figure S3B in Supplementary Material), PBMCs were stimulated with different combinations of cytokines or PHA for 5 days to compare the proliferative capacities of CD161^+^ and CD161^−^ NK cells. Strikingly, CD161^+^ NK cells divided more extensively than CD161^−^ NK cells when stimulated with IL-2 or IL-15, either alone, together, or combined with IL-12, as well as PHA (Figures 3A,B). There was no significant difference in the expression of CD69 in response to IL-2, IL-15, IL-18, or PHA, suggesting that the reduced proliferation in CD161^−^ NK cells is not due to their inability to respond to the cytokines. In all other conditions, expression of the activation marker CD69 was consistently higher in the CD161^+^ NK cell subset, reaching significance for all conditions with IL-12 or IL-15 (Figure 3C). Furthermore, there was no significant difference in phosphatidylserine staining, a marker of apoptosis, between CD161^+^ and CD161^−^ NK cells after culture with IL-2 or IL-15 (Figure 3D), suggesting that the lower proliferative response of the CD161^−^ NK cells was not due to higher rates of activation-induced cell death in this subset. The higher proliferation observed from CD161^+^ NK cells was also confirmed using FACS-sorted CD161^+^ and CD161^−^ NK cells (Figures 3E,F). Interestingly, proliferating NK cells downregulated CD161 expression with increasing rounds of division (Figure S4A in Supplementary Material). Together, these results suggest that CD161^+^ NK cells have a greater proliferative capacity compared to CD161^−^ NK cells.

Of note, CD57 expression on NK cells (33, 34) has been demonstrated to identify cells with low proliferative capacity. Analysis of CD161 and CD57 expression on NK cells proliferating following IL-15 stimulation for 5 days, however, showed that within both CD57^−^ and CD57^+^ populations, the CD161^+^ NK cells showed greater dilution of the CTV dye (Figures 3G,H). This shows that CD161 expression correlates with the proliferative capacity of NK cells, independently of CD57.

### CD161^+^ NK Cells Are Enriched for CD103-Expressing Cells in the Inflamed Gut

**Figure 4:**
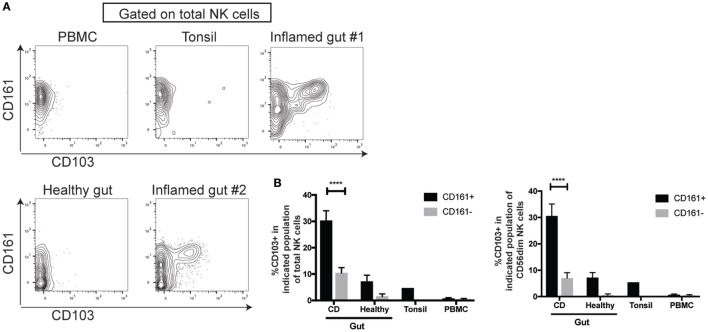
CD161^+^ NK cells are enriched for CD103-expressing cells in the inflamed gut. **(A,B)** FACS plots **(A)** and bar graph **(B)** showing CD103 expression on indicated natural killer (NK) cell populations isolated from the periphery (*n* = 5), tonsil (*n* = 1), or the lamina propria, either inflamed from IBD patients (*n* = 7) or non-inflamed lamina propria (*n* = 4) as analyzed by mass cytometry. FACS plots gated on total CD3^−^CD56^+^ NK cells. IBD, inflammatory bowel disease.

As CD161^int^CD8^+^ T cells are enriched in the gut and express CD103 (28), an integrin associated with tissue-residency, we addressed whether CD161^+^ NK cells showed a similar association. Lamina propria lymphocytes were isolated from inflamed and non-inflamed areas of the small intestine from patients with IBD [mostly Crohn’s disease (CD)] and analyzed by mass cytometry. We found a significantly higher expression of CD103 on CD161^+^ compared to CD161^−^ NK cells in the inflamed gut (Figure 4A), both among total NK cells and CD56^dim^ NK cells (Figure 4B). CD103 expression was found to be at a lower frequency in healthy gut and tonsil, but not found on NK cells from the periphery. We also observed higher proportion of CD161^+^ NK cells expressing the tissue-residency markers CD103, CD69, and integrin-β7 (Figure S5 in Supplementary Material) in the inflamed intestinal lamina propria of IBD patients.

Thus CD161 expression marks NK cells that share transcriptional and functional features with CD8^+^ T cells that also express CD161 and are enriched for CD103-expressing cells in the inflamed lamina propria.

### High-Dimensional Analysis Delineates NK Cell Populations in CMV+ and CMV− Donors

**Figure 5:**
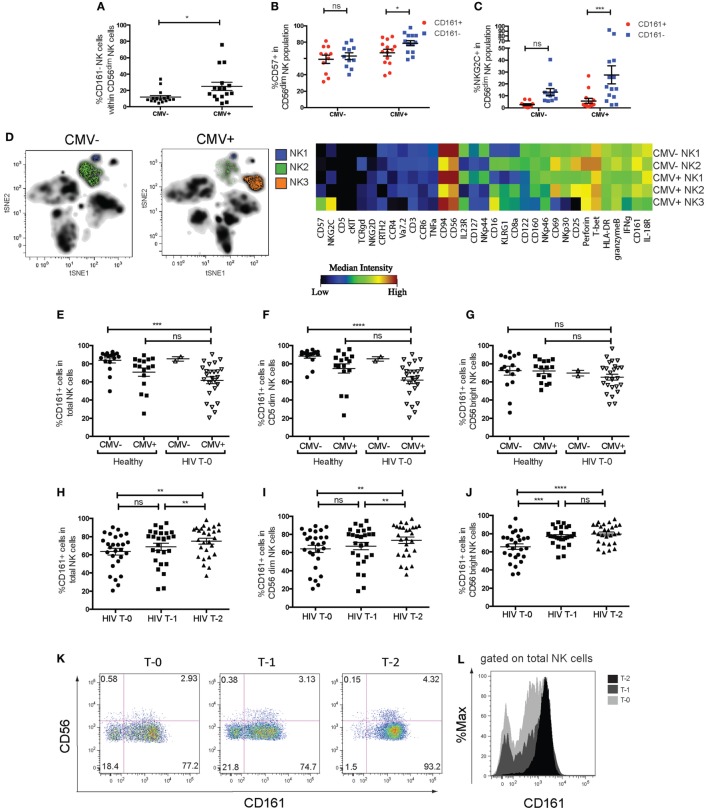
Changes in CD161 expression in cytomegalovirus (CMV) and human immunodeficiency virus (HIV) infection. **(A)** Frequencies of CD161^−^ NK cells within the CD56^dim^ natural killer (NK) cell population within CMV+ (*n* = 16) and CMV− (*n* = 16) individuals. Data combined from four independent experiments. **(B,C)** Frequencies of **(B)** CD57^+^ cells or **(C)** NKG2C+ cells in CD161^+^ or CD161 population of CD56^dim^ NK cells, in CMV+ and CMV− donors. *N* = 14 for CMV+, *n* = 11 for CMV− individuals. **(D)** Peripheral and cord blood samples were stimulated with IL-12+IL-18 overnight and acquired by mass cytometry. **(H)** t-distributed stochastic neighbor embedding analysis was performed on CMV+ (*n* = 5) and CMV− donors (*n* = 6). The median intensities of each marker were calculated for the NK cell populations identified (left) and plotted as a heatmap (right). **(E–L)** The frequency of CD161-expressing NK cells in patients with chronic-stage HIV infection from the Swiss HIV cohort study, who were followed for 2 years of anti-retroviral therapy (ART), with samples taken prior to the start of the treatment (T-0), at 1 year (T-1), and 2 years (T-2) into treatment. Patients were tested for CMV seropositivity prior to the start of treatment (*n* = 27, Table S2 in Supplementary Material). The frequency of CD161^+^ NK cells within **(E)** total NK cells (CD3^−^CD56^+^), **(F)** CD56^dim^ NK cells, and **(G)** CD56^bright^ NK cells is shown for HIV patients at T-0 (*n* = 25 for CMV+, *n* = 2 for CMV−), compared to CMV+ (*n* = 16) and CMV− (*n* = 16) healthy donors. **(H–J)** Frequency of CD161^+^ NK cells within HIV patients followed for 2 years of ART. CD161^+^ cells within **(H)** total NK cells, **(I)** CD56^dim^ NK cells, and **(J)** CD56^bright^ NK cells are shown for HIV patients at T-0, T-1, and T-2. **(K)** Representative staining showing CD161 expression on total NK cells from one patient at T-0 (left), T-1 (center), and T-2 (right). **(L)** Histogram overlaying CD161 expression on total NK cells from one patient.

Cytomegalovirus infection has been demonstrated to accelerate the differentiation of NK cells, leading to the persistence of terminally differentiated NK cells expressing the activating heterodimer NKG2C/CD94 and CD57 (18, 35). Significant downregulation of CD161 on these cells has been reported (17, 20, 36). Confirming these findings, we found that CMV+ individuals had a significantly greater frequency of CD161^−^ cells within their CD56^dim^ NK cell population (Figure 5A), but not CD56^bright^ NK cells (data not shown). Furthermore, there was a higher frequency of CD57^+^ and NKG2C+ cells (Figures 5B,C) within the CD161^−^ CD56^dim^ NK cell population in CMV+ but not CMV− individuals, as expected (17, 20, 36).

Peripheral blood mononuclear cells from CMV+ and CMV− adults, as well as cord blood samples, were stimulated with IL-12 and IL-18 and the expression of 39 markers was simultaneously assessed by mass cytometry (Table S3 in Supplementary Material). t-SNE analysis (30, 37) of the expression of 36 parameters on live lymphocytes generated a two-dimensional composite map that clearly delineates cell subsets as distinct niches, or clusters (Figure 5D). NK cell clusters were further analyzed to generate a comprehensive overview of marker expression in each cluster. For the three NK cell clusters in one representative donor identified in Figure 5D, the median intensities of each marker was quantified and summarized using a heatmap illustration. This shows a clearly lower expression of NKp30, CD25, CD69, IFNγ, CD160, and to a lesser extent, CD161 and IL-18R in the CMV+ donor-specific NK3 cluster, while they expressed higher levels of NKG2C, CD57, and CD16.

t-Distributed stochastic neighbor embedding analysis of the expression of these parameters on NK cells from all donors identified five distinct clusters (Figure S6 in Supplementary Material). Interestingly, two of these NK cell clusters were high in NKG2C expression (clusters 1 and 2), and while one of these NKG2C^+^CD161^−^IFNγ^−^ clusters (cluster 1) was only present in CMV+ individuals, cluster 2 was present in CMV− adults but not cord blood, suggesting that CMV may induce the expansion of these NKG2C^+^CD161^−^IFNγ^−^ cells (cluster 2), which further differentiates into cluster 1. Thus, CD161 expression marks CD56^dim^NK cells that have retained their responsiveness to innate cytokines; an ability that is lost upon CMV-induced terminal differentiation.

### CD161^+^ NK Cells Are Depleted in CMV-Seropositive HIV Donors and Recover with ART

We next wanted to investigate whether CD161 expression changes in other viral diseases. CD161 expression on NK cells has been reported to be reduced in the periphery of chronic HIV patients (13, 38, 39). To investigate whether this subset recovers in patients receiving ART, the frequency of CD161^+^ NK cells in 27 patients with chronic HIV infection from the SHCS were followed for 2 years of ART. Samples were taken prior to the start of treatment (T-0), at 1 year (T-1), and 2 years (T-2) into treatment and analyzed by flow cytometry in a blinded manner.

Previous findings that the frequency of CD161^+^ NK cells were dramatically lower in HIV patients was initially confirmed by comparing the frequency of CD161^+^ NK cells in these HIV patients to that in healthy controls (Figure S7 in Supplementary Material). However, our initial comparison did not take into account the CMV seropositivity of the “healthy” controls. CMV infection and reactivation is common in immunosuppressed HIV patients (40, 41), and indeed 25 out of 27 patients were CMV seropositive in the SHCS cohort. In order to control for the expansion of the CD161^−^ NK cells that occurs in CMV+ donors, the frequency of CD161^+^ NK cells in CMV+ HIV patients and CMV+ healthy donors were compared. We found that within total NK cells (Figure 5E) and CD56^dim^ NK cells (Figure 5F), there was a significantly lower frequency of cells expressing CD161 in CMV+ HIV patients, compared to healthy CMV− donors, but not compared to healthy CMV+ donors. There was no significant difference in CD161 expression in CD56^bright^ NK cells between healthy donors and HIV patients, regardless of CMV status (Figure 5G). This suggests that CD161 downregulation on NK cells in HIV patients may be associated with their CMV seropositive status, due to the lower expression of CD161 on CD56^dim^ NK cells in CMV+ individuals. Indeed, the two CMV seronegative HIV patients had a high expression of CD161, with 83 and 87.8% of CD56^dim^NK cells expressing CD161, respectively, compared to a mean of 61.90 ± 3.84% of CD56^dim^NK cells from CMV+ HIV patients expressing CD161.

Next, we investigated whether CD161 expression recovers with successful ART treatment. Surprisingly, a comparison of CD161 expression in total, CD56^dim^, and CD56^bright^ NK cells (Figures 5H–J) showed that in all populations, there was a significant increase in the frequency of cells expressing CD161 with treatment (T-0 vs. T-2; Figures 5K,L). Interestingly, there was a small but significant correlation between CD4 counts and frequency of cells expressing CD161 in both CD56^bright^ and CD56^dim^NK cells (Figures S7C,D in Supplementary Material) in these patients after, but not before, 2 years of ART treatment.

## Discussion

In this study, the transcriptional signature and functional capacity of human CD161^+^ and CD161^−^ NK cells was investigated. We show that CD161 marks cytokine-responsive NK cells, independently of CD57 or CMV status, sharing transcriptional and functional features with T cells expressing CD161. These findings are important given the recent observation that CD161 was the best prognostic marker expressed by tumor-associated leukocytes across 39 different malignancies (42). Furthermore, in contrast to “adaptive” NK cells, which are highly responsive to antibody-opsonized target cells but not to cytokines or tumor target cells (17), these pro-inflammatory, “innate” NK cells have the potential to play a role in the inflamed gut.

CD161 is expressed early in NK cell development, where it may facilitate cross-talk of NK cell precursors with cells within the bone marrow, and is involved in CXC8 release (6–8). Within the periphery, cross-linking of CD161 leads to an increase in IFNγ expression and inhibition of NK cell (43–47). In this study, under certain stimulatory conditions, CD161 expression decreases with proliferation (Figure S4A in Supplementary Material); raising the question if CD161- NK are derived from highly activated NK cells. However, a complete loss of CD161 was not observed, even on those cells that had under gone the greatest rounds of proliferation, suggesting that additional factors would be required.

The most striking functional difference between CD161^+^ and CD161^−^ NK cells was their capacity to respond to pro-inflammatory cytokines, particularly within CD56^dim^ NK cells. Thus, CD161 identifies NK cells that have retained the ability to respond to IL-12 and IL-18 during differentiation, expressing highest levels of IFNγ, NKp30, CD160, CD25 (part of the high-affinity IL-2 receptor) and CD69 following stimulation. This is in line with recent studies showing that reduced expression of the transcription factor promyelocytic leukemia zinc finger (PLZF) in NKG2C^+^CD57^++^ “adaptive” NK cells in CMV leads to downregulation of CD161, IL-12R and IL-18R in these cells (17, 48). Here we have found using multidimensional analysis that this functional association between CD161 and IL-12 and IL-18 responsiveness was preserved in both CMV+ donors and CMV− donors, and this functional correlation was also independent of CD57 expression that marks mature NK cells (33). Thus, CD161 is a marker of pro-inflammatory NK cell function, independent of CMV-induced differentiation.

Whether NK cells lose CD161 in a linear fashion as they mature is unclear. All NK cells expressed CD161 at a high level in cord blood, but a distinct CD161^−^ NK population was present in 24-month-old samples, suggesting that CD161^−^ NK cells expand early in life. Interestingly, not all the properties of CD161+ NK cells are consistent with these cells being more immature than the CD161^−^ NK cells. For instance, CD161^−^ cells are present in the CD56bright NK cell population which are considered to be more immature than CD56dim NK cells, and there was no difference in CD57 expression between CD161+ and CD161^−^ cells in CMV-negative donors. Instead CD161 expression may be linked with education which is disassociated from NK cell maturation (34). CD161 ligation has been shown to inhibit NK cell cytotoxicity (4, 5). The downmodulation of the inhibitory molecule CD161 may enhance the ability of NK cells to kill infected or transformed cells, but would also result in unchecked cytotoxicity that could be harmful to “self.” As NK cells are licensed through ligation of KIRs by self-specific HLA ligands (49, 50) it is tempting to speculate that downregulation of CD161 may be associated with increased education and expression of other inhibitory receptors that recognize classical MHC class I molecules. Indeed, NKG2C^+^CD57^+^ cells have clonal expression pattern of inhibitory KIRs specific for self-HLA class I molecules (20, 43). A population of NKG2C-negative NK cells expressing inhibitory self-KIRs can also expand in CMV+ individuals (43–45). As CMV induces the downregulation of MHC class I molecules, except for HLA-E, cells expressing inhibitory self-KIRs and NKG2C (which binds HLA-E) may selectively expand (44). Alternatively, as CD161 expression is controlled by the chromatin modifying transcription factor PLZF (17, 51), its expression may be linked to factors other than maturation such as epigenetic modification.

Previous studies have shown that, in the context of HCMV, IL-15-induced NK cell proliferation is dependent on receptor signaling, in particular through NKG2C (52). We confirm these findings and show that this is restricted to the CD161- population (*n* = 1, Figure S4C in Supplementary Material). Interestingly, cytokine-induced memory-like NK cells can be induced by pre-activation with IL-12, IL-15, and IL-18 (53), cytokines that CD161^+^ NK cells are most responsive to. These cytokines induce the upregulation of CD25, which allows these cells to respond to IL-2 produced by memory T cells. Whether these memory-like NK cells are indeed derived from CD161^+^ NK cells, perhaps following cytokine-induced expansion and downregulation of CD161 (Figure S4A in Supplementary Material) requires further investigation. This would be important in understanding the pathway leading to the generation of memory and provide a useful marker in the ongoing therapeutic application of cytokine-induced memory-like NK cells in hematologic malignancies (53).

We observed a higher proportion of CD161^+^ NK cells expressing the tissue-residency markers CD103, CD69, and integrin-β7 (Figure S5 in Supplementary Material) in the inflamed intestinal lamina propria of IBD patients. This is reminiscent of the increased frequency of CD103^+^ intraepithelial (ie)ILC1 cells in the epithelia of CD patients (54). Interestingly these cells have recently been redefined as NK cells that reside in mucosal and non-mucosal pathological tissues (55). Indeed, consistent with Simoni and colleagues’ findings, the CD103^+^ NK cells identified in our study expressed CD56, CD94, Eomes, perforin, and lacked NKp44 or CD127 expression (Figure S5 in Supplementary Material). These ieILC1-like cells are enriched within the CD161^+^ NK cell population in the lamina propria of IBD patients. Given the increased presence of ieILC1-like cells in inflamed intestinal tissues, as well as non-mucosal pathological tissues (e.g., tumors), the CD161^+^ subset of these cells may contribute to disease pathogenesis because of their ability to respond to the abundant IL-12 and IL-18 that characterizes CD and secrete IFNγ (56). Indeed, IFNγ-producing ieILC1 cells have been shown to contribute to the development of colitis in a murine model of IBD (54).

In the present study of HIV-infected patients, CD161 expression in CD56^dim^ NK cells was significantly reduced compared to healthy CMV− donors, but not compared to healthy CMV+ donors. Although only two HIV patients were CMV seronegative, both donors had high expression of CD161, which did not change with ART (data not shown). Therefore, CD161 downregulation may be associated with CMV reactivation or re-infection due to progressive immunodeficiency during HIV infection, as has been demonstrated for increased NKG2C expression in HIV patients (57, 58). The loss of the CD161^+^ NK cell subset in HIV-infected patients may potentially lead to cells having a reduced proliferative capacity in response to homeostatic cytokines as well as a reduced capacity to secrete IFNγ in response to innate cytokines (59). Prior infection with CMV is a risk factor for a low CD4 count, progression to AIDS and mortality in HIV patients (40). Whether CMV-driven differentiation, and possibly exhaustion, of NK cells reduces their ability to mediate HIV control will be important to investigate, given recent evidence of their role in elite controllers (60) and reducing the size of the viral reservoir (61).

In summary, we have shown that CD161 is a marker of NK cell function, particularly marking pro-inflammatory NK cells, which is expressed on all NK cells in cord blood, downregulated upon proliferation, and lost upon CMV-induced terminal differentiation. CD161 expression on CD56^dim^ NK cells marks cells that have retained the capacity to respond to inflammatory cytokines alone. CD161, thus, identifies “innate” cells within both NK cells and CD161bright T cell family, previously described, with shared innate-type responses that are independent of receptorligation (25).

## Ethics Statement

Adult and cord blood samples were collected after ethical approval by the Central Office for Research Ethics Committees (COREC, local research ethics committee Oxford), reference number COREC 04.OXA.010. The NHS Research Ethics System provided ethical approval for the Oxford IBD Cohort study (reference numbers 09/H0606/5 for IBD patients and 11/YH/0020 and 16/YH/0247 for controls). All patients from the studies above provided their informed written consent. The collection of blood samples for the 24-month-old study cohort was approved by the Human Ethics Committee at Huddinge University Hospital, Stockholm, reference code 75/97, 331/02, and the parents provided their informed verbal consent. No written documentation of the participants informed approval was required, which was agreed to by the Human Ethics Committee and was according to the regulations at the time of the initiation of the study. The SHCS was approved by the local ethical committees of the participating centres: Kantonale Ethikkommission Zürich (KEK-ZH-NR: EK-793); Ethikkommission beider Basel (“Die Ethikkommission beider Basel hat die Dokumente zur Studie zustimmend zur Kenntnis genommen und genehmigt.”); Kantonale Ethikkommission Bern (21/88); Comité departmental d’éthique des specialités médicales es de médecine communautarie et de premier recours, Hôpitaux Universitaires de Genève (01–142); Commission cantonale d’éthique de la recherche sur l’être humain, Canton de Vaud (131/01); Comitato etico cantonale, Repubblica e Cantone Ticino (CE 813); Ethikkommission des Kantons St. Gallen (EKSG 12/003), and written informed consent was obtained from all participants.

## Author Contributions

AK designed and performed experiments, analyzed data, and wrote the manuscript. CC and BW performed experiments. YS and EN helped with mass cytometry experiments and analysis. AG, SB, ES-E, CA-C, CT, HG, and NK provided samples. See Supplementary Materials for full list of SHCS and Oxford IBD Cohort investigators. CW helped design experiments. PK supervised research work and data analysis. Dr. Walker contributed by providing samples, experimental design, and important intellectual content.

## Conflict of Interest Statement

The authors declare that the research was conducted in the absence of any commercial or financial relationships that could be construed as a potential conflict of interest.
