# Supplementary material for: CD161 Defines a Functionally Distinct Subset of Pro-Inflammatory Natural Killer Cells
Source: Front Immunol. 2018 Apr 9;9:486. doi: 10.3389/fimmu.2018.00486 (PMC5900032; doi:10.3389/fimmu.2018.00486)
Supplement: Supplementary file 1 [file table_1.PDF]

Supplementary Table 1. Clinical characteristics of inflammatory bowel disease patients. CD=Crohn's disease, UC=ulcerative colitis, LPMC=lamina proprial mononuclear cells.

ID	diagnosis	resected tissue	cell type	gender	age	treatment	behaviour	location	date of diagnosis	disease duration (y)
IBD_1	CD	ileum	LPMC	M	30	nil	fistulating	ileal	2013	2
IBD_2	UC	rectum	LPMC	M	52	nil	N/A	rectum	2010	5
IBD_3	CD	right colon	LPMC	M	28	nil	stricturing	ileo-colonic	2005	10
IBD_4	CD	right colon	LPMC	F	37	adalimumab (anti-TNF)	stricturing	ileo-colonic	2002	13
IBD_5	CD	ileum	LPMC	M	28	nil	fistulating	ileal	2015	0
IBD_6	CD	colon	LPMC	M	28	nil	fistulating	ileal	2015	0
IBD_7	CD	ileum	LPMC	M	42	nil	fistulating	ileal	2006	9
