# Supplementary material for: CD161 Defines a Functionally Distinct Subset of Pro-Inflammatory Natural Killer Cells
Source: Front Immunol. 2018 Apr 9;9:486. doi: 10.3389/fimmu.2018.00486 (PMC5900032; doi:10.3389/fimmu.2018.00486)
Supplement: Supplementary file 2 [file table_2.PDF]

	Treatment time point		
	Pre treatment	1 year post treatment	2 years post treatment
**n**	27	27	27
**Gender**<br>Male<br>Female	 24<br>3		
**Age**<br>Median (IQR)	 40 (34.0, 45.0)		
**CD4 count (cells/ml)**	202 (185,299)	414 (333, 569)	515 (405, 620)
**Viral load (log10 cpm)**<br>Median (IQR)	 4.64 (4.13, 4.98)	 0 (0,0)	 0 (0,0)
**Time since diagnosis (months)**<br>Median (IQR)	10 (2,86)		
**CMV seropositive**	25	not tested	not tested

Supplementary Table 2. Clinical characteristics of treatment cohort from Swiss HIV Cohort Study. Modified from Cosgrove *et al.*, 2013.
