# Supplementary material for: CD161 Defines a Functionally Distinct Subset of Pro-Inflammatory Natural Killer Cells
Source: Front Immunol. 2018 Apr 9;9:486. doi: 10.3389/fimmu.2018.00486 (PMC5900032; doi:10.3389/fimmu.2018.00486)
Supplement: Supplementary file 3 [file table_3.PDF]

Supplementary Table 3. Mass cytometry panel for experiments in Figure 5.

Atomic mass	Markers
103	Barcode (BC)
104	BC
105	BC
106	BC
108	BC
110	BC
113	BC
89	CD45
112/114	CD14
115	CD57
141	HLA-DR
142	NKG2D
143	IFNγ
144	CD16
145	CD69
146	CD8
147	TNFα
148	CCR4
149	CCR6
150	KLRG1
151	CD5
152	CD122
154	CXCR6
155	T-bet
156	FCεER1a, CD11c, CD19, CD123
158	CD56
159	CD161
160	NKp44
161	NKp30
162	Vα7.2
163	CD127
164	Granzyme B
165	IL-18R
166	NKp46
167	c-Kit-APC
168	CD3
169	CD25
170	NKG2C
171	CRTH2-FITC
172	CD94
173	IL-23R-biotin
174	CD160
175	Perforin
176	γδ TCR-PE
191/193	DNA
195	Cisplatin (Live/dead)
