# Supplementary material for: CD161 Defines a Functionally Distinct Subset of Pro-Inflammatory Natural Killer Cells
Source: Front Immunol. 2018 Apr 9;9:486. doi: 10.3389/fimmu.2018.00486 (PMC5900032; doi:10.3389/fimmu.2018.00486)
Supplement: Supplementary file 5 [file image_1.PDF]

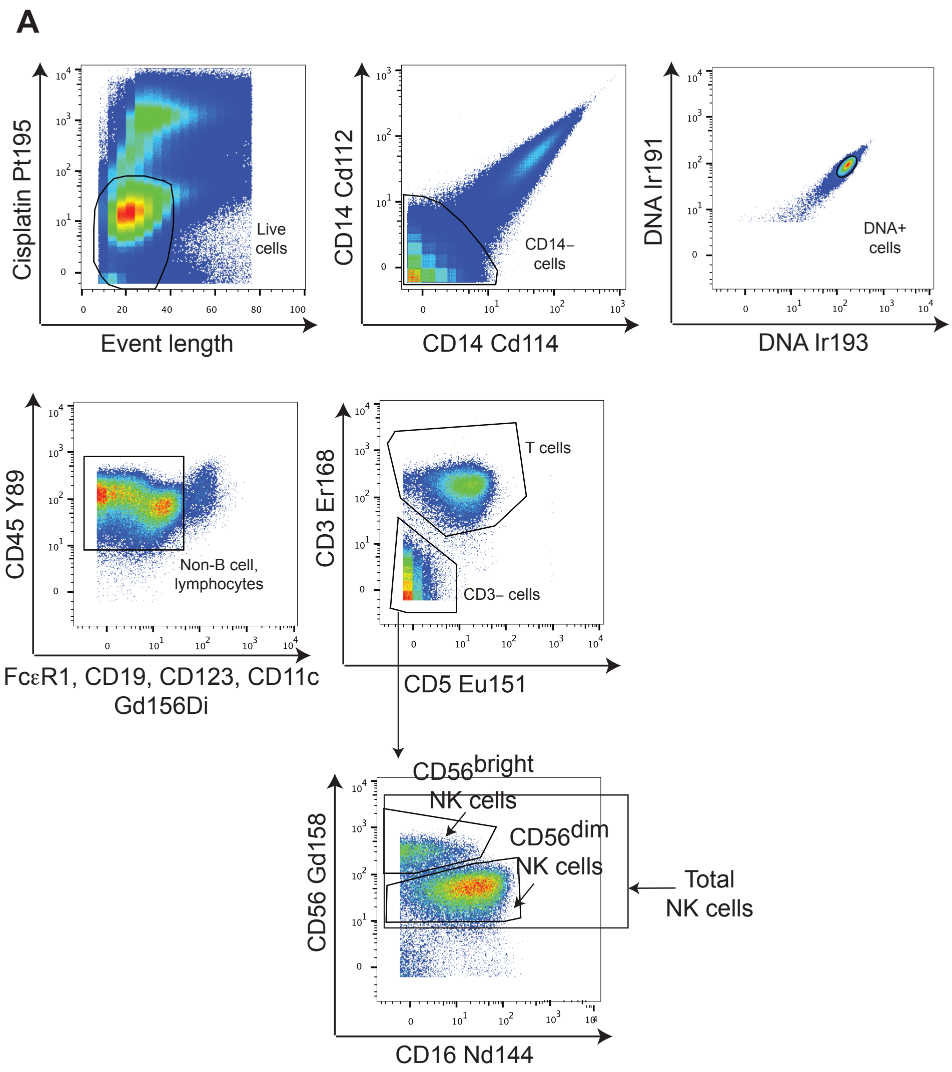

Supplementary Figure 1. Gating strategy for identifying NK cells by mass cytometry. Monocyte, B cell, and CD4+ T cell-depleted peripheral blood and cord blood samples were stimulated with IL-12+IL-18 overnight and acquired by mass cytometry. A) Live cells were identified as Cisplatin- and DNA+ cells. Lymphocytes were further identified by the expression of CD45, and monocytes (CD14+), mast/basophils (FcεR1+), plasmacytoid DCs (CD123+), myeloid DCs (CD11c+) were excluded. B cells (CD19+) were also excluded from the analysis. NK cells were gated as CD3-CD5-CD56+ cells.
