# Supplementary material for: CD161 Defines a Functionally Distinct Subset of Pro-Inflammatory Natural Killer Cells
Source: Front Immunol. 2018 Apr 9;9:486. doi: 10.3389/fimmu.2018.00486 (PMC5900032; doi:10.3389/fimmu.2018.00486)
Supplement: Supplementary file 6 [file image_2.PDF]

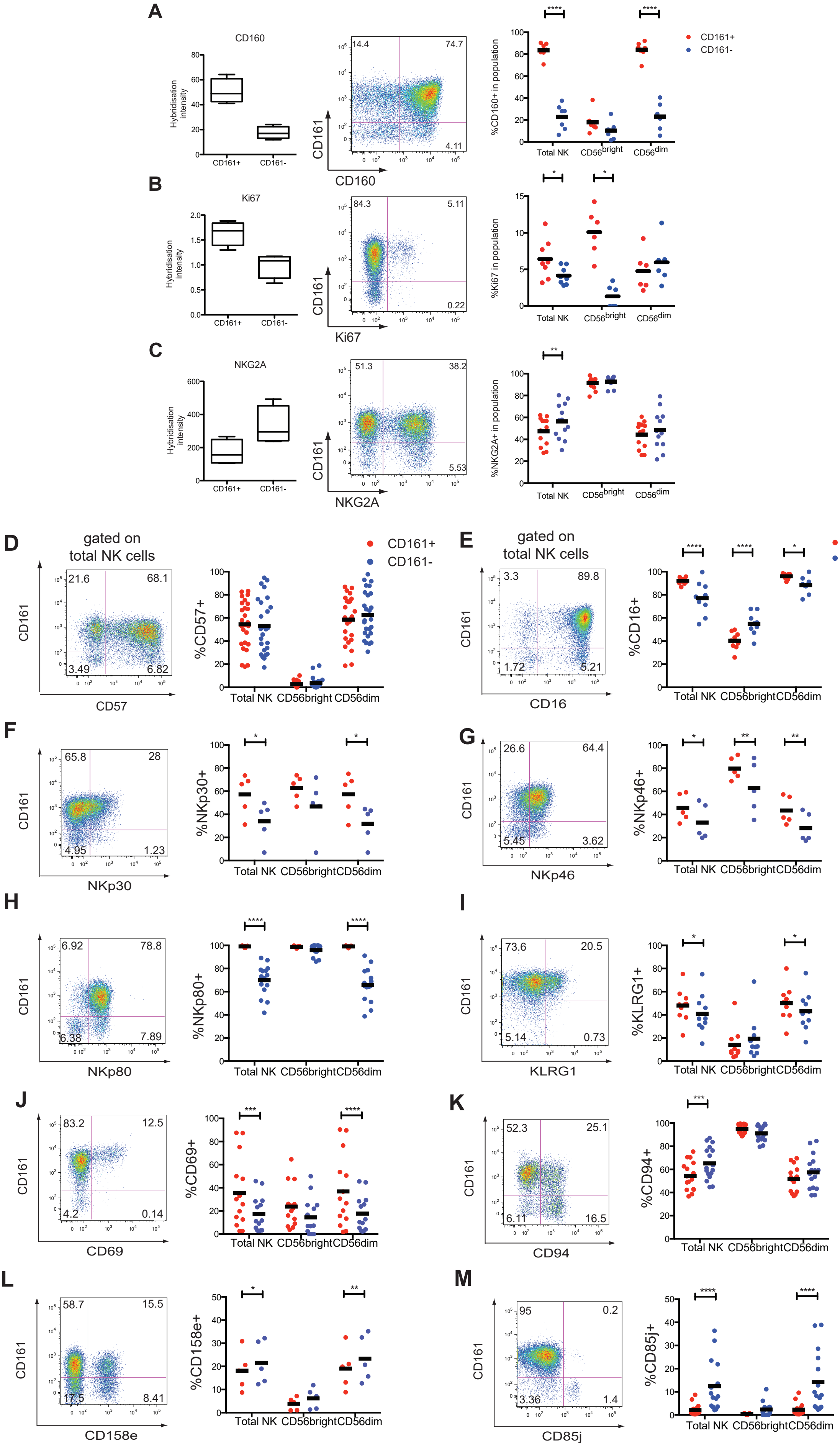

Supplementary Figure 2. Analysis of phenotypic marker expression in CD161+ and CD161- NK cells. A-C) Expression of CD160 (A), Ki67 (B), NKG2A (C) by gene array (left), representative plot by flow cytometry (centre) and frequencies of CD161+ and CD161− cells expressing the indicated marker within total NK, CD56bright and CD56dimNK cells. D-M) CD161+ and CD161−NK cells from adult peripheral blood were assessed for the expression of indicated markers. Representative plots, gated on total NK cells (CD3−CD56+) (left), and frequencies of receptor-positive cells within the CD161+ and CD161− fractions of indicated NK cell populations (right) are shown for 5-26 healthy donors. Expression of D) CD57 (n=26), E) CD16 (n=9), F) NKp30 (n=5), G) NKp46 (n=5), H) NKp80 (n=15), I) KLRG1 (n=10), J) CD69 (n=14), K) CD94 (n=17), L) CD158e (n=5), M) CD85j (n=15) is shown. In all graphs, CD161+ cells are shown in red, CD161− cells are shown in blue. CD161+ cells are compared to CD161− cells within each population of NK cells.
