# Supplementary material for: CD161 Defines a Functionally Distinct Subset of Pro-Inflammatory Natural Killer Cells
Source: Front Immunol. 2018 Apr 9;9:486. doi: 10.3389/fimmu.2018.00486 (PMC5900032; doi:10.3389/fimmu.2018.00486)
Supplement: Supplementary file 7 [file image_3.PDF]

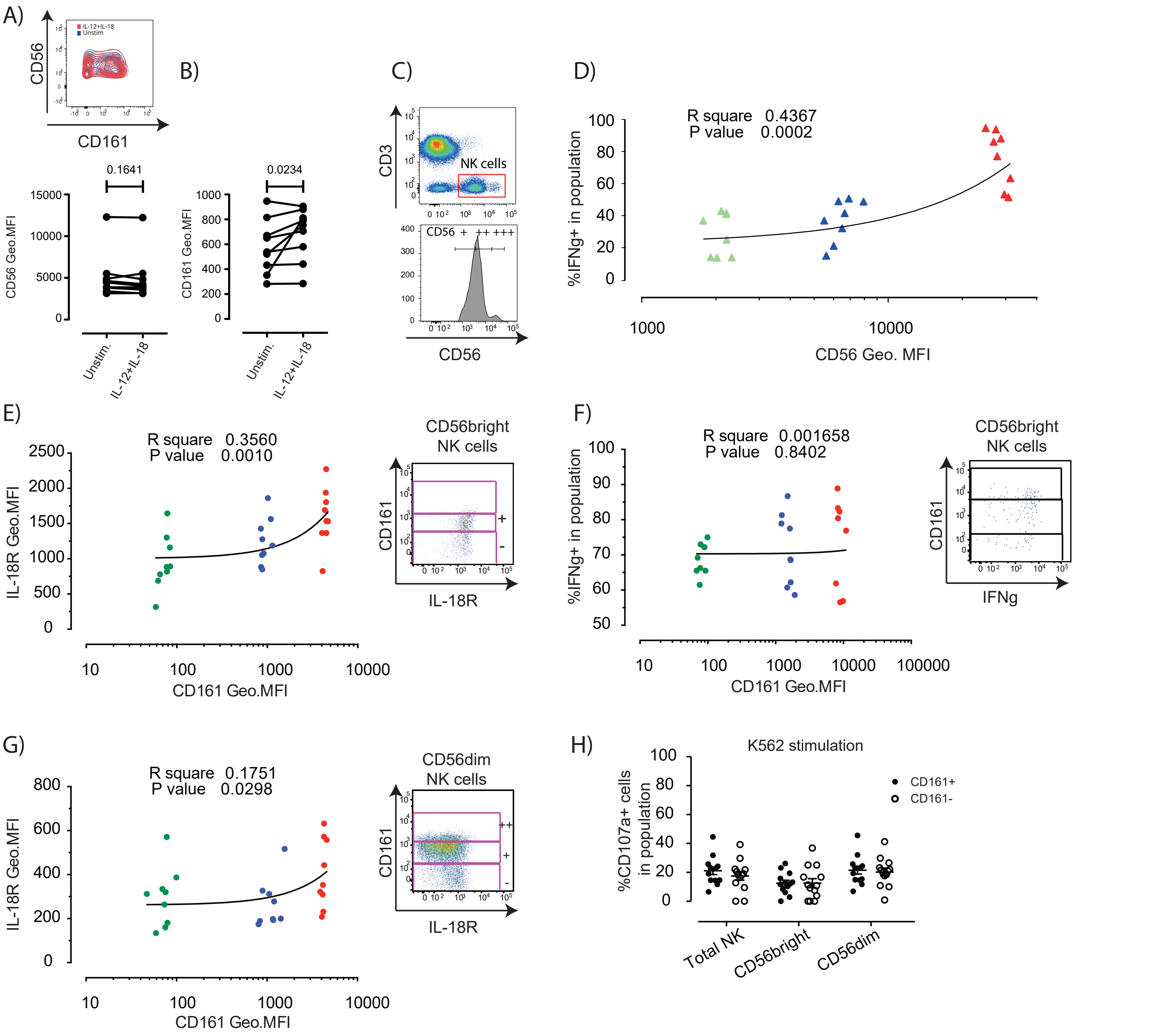

Supplementle figure 3:CD161 and CD56 expression correlate with IL-12+IL-18 responsiveness. A-B) Top, representative contor CD56 vs CD161 plot of NK cells either unstimulated, blue, or stimulated with IL-12 and IL-18, red. Below, CD56 and CD161 expression after overnight stimulation with IL-12+IL-18 stimulation. C) Arbitrary division of the NK CD56+ population into low (+), intermediate (++), and high (+++) expression levels. D) Correlation between CD56 expression, divided as in (C), and IFNgamma expression. E) Correlation between CD161 and IL18receptor expression in the CD56bright population, with a representative FACS plot to the right. F) Correlation between CD161 expression and IFNgamma expression in the CD56bright population, with a representative FACS plot to the right. G) Correlation between CD161 expression and IL-18 receptor expression in the CD56dim population, with a representative FACS plot to the right. H) CD107a surface expression on NK cell populations, CD161+ or CD161-, after overnight co-culture with K562 target cells.
