# Supplementary material for: CD161 Defines a Functionally Distinct Subset of Pro-Inflammatory Natural Killer Cells
Source: Front Immunol. 2018 Apr 9;9:486. doi: 10.3389/fimmu.2018.00486 (PMC5900032; doi:10.3389/fimmu.2018.00486)
Supplement: Supplementary file 8 [file image_4.PDF]

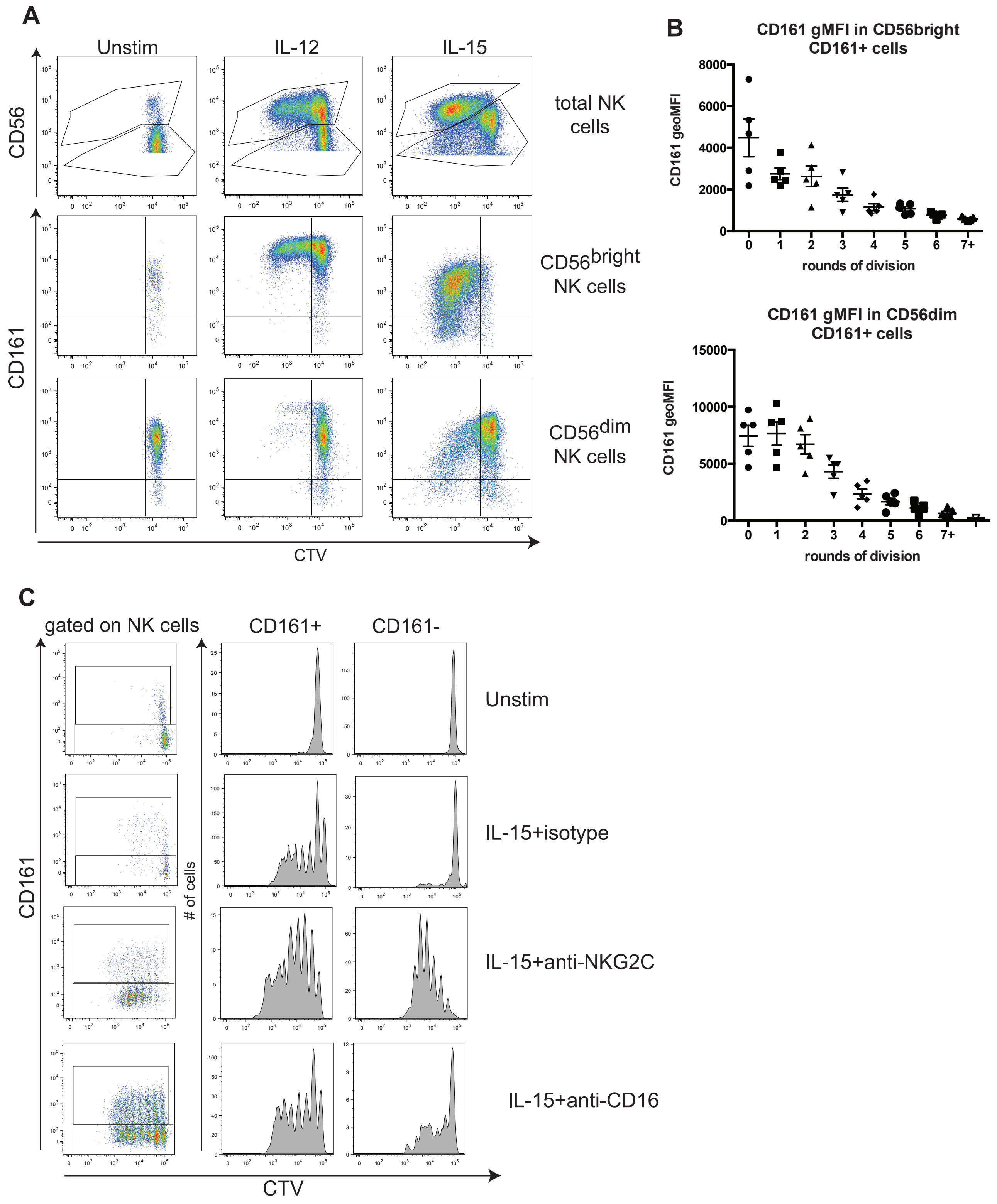

Supplementary Figure 4. Downregulation of CD161 on proliferating NK cells. A) Expression of CD161 on NK cells proliferating in response to IL-12 and IL-15 following stimulation for 5 days. B) Expression of CD161 as measured by gMFI with increasing rounds of divisions in response to IL-15 stimulation in CD56bright CD161+ NK cells (top) and CD56dim CD161+ NK cells (bottom) (n=5). C) CTV-labeled NK cells were stimulated with IL-15 and plate-bound anti-NKG2C or anti-CD16 antibody, or isotype control for 5 days (n=1).
