# Supplementary material for: CD161 Defines a Functionally Distinct Subset of Pro-Inflammatory Natural Killer Cells
Source: Front Immunol. 2018 Apr 9;9:486. doi: 10.3389/fimmu.2018.00486 (PMC5900032; doi:10.3389/fimmu.2018.00486)
Supplement: Supplementary file 9 [file image_5.PDF]

A

B

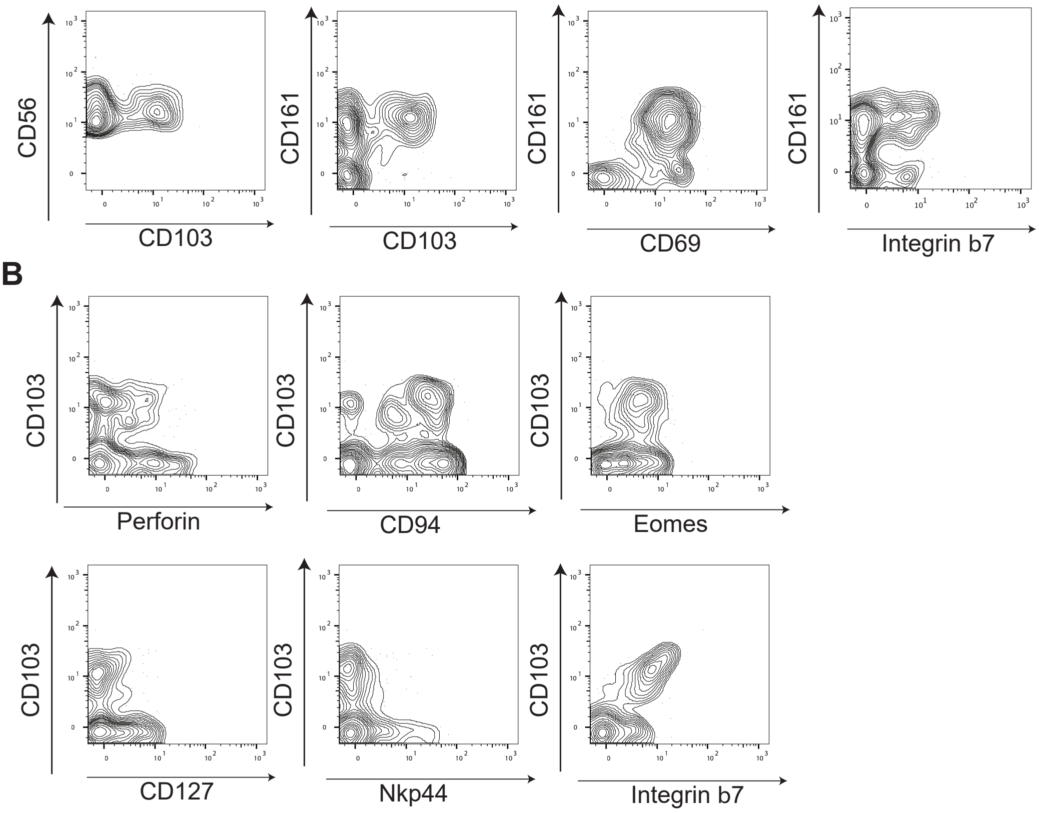

Supplementary Figure 5. Phenotype of CD103+ ILC1-like NK cells in the lamina propria of inflammatory bowel disease (IBD) patients. NK cells were gated as CD3-CD56+ cells and the expression of the indicated markers against CD103 or CD161 are shown from one representative IBD patient. A) Expression of CD103 on total NK cells, against CD56, or CD161, or expression of CD69 or Integrin b7 against CD161. (from left to right). B) Expression of the indicated markers against CD103 on total NK cells, showing CD103+ cells are perforin+, CD94+, Eomes+, CD127-, NKp44-, and Integrin b7+.
