# Supplementary material for: CD161 Defines a Functionally Distinct Subset of Pro-Inflammatory Natural Killer Cells
Source: Front Immunol. 2018 Apr 9;9:486. doi: 10.3389/fimmu.2018.00486 (PMC5900032; doi:10.3389/fimmu.2018.00486)
Supplement: Supplementary file 10 [file image_6.PDF]

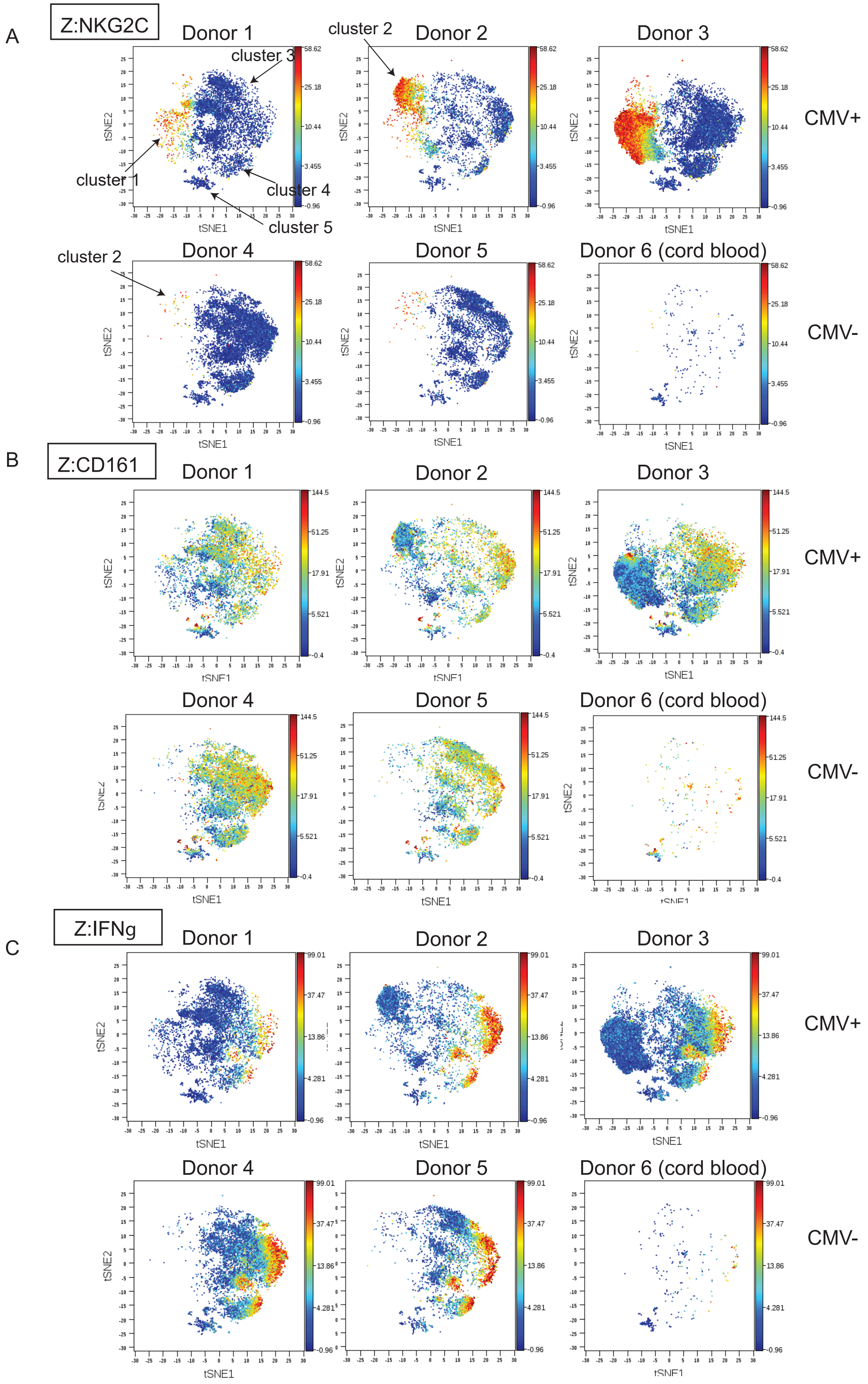

Supplementary Figure 6. t-SNE analysis gating on NK cells (CD3-CD5-CD56+ cells) only.
The analysis delineates 5 distinct clusters of NK cells, highlighted by the arrows. Plots are coloured by the expression level of the marker indicated by Z. A) Colouring of t-SNE plots according to NKG2C expression levels. B) Colouring of t-SNE plots according to CD161 expression levels. C) Colouring of t-SNE plots according to IFNγ expression levels.
