# Supplementary material for: CD161 Defines a Functionally Distinct Subset of Pro-Inflammatory Natural Killer Cells
Source: Front Immunol. 2018 Apr 9;9:486. doi: 10.3389/fimmu.2018.00486 (PMC5900032; doi:10.3389/fimmu.2018.00486)
Supplement: Supplementary file 11 [file image_7.PDF]

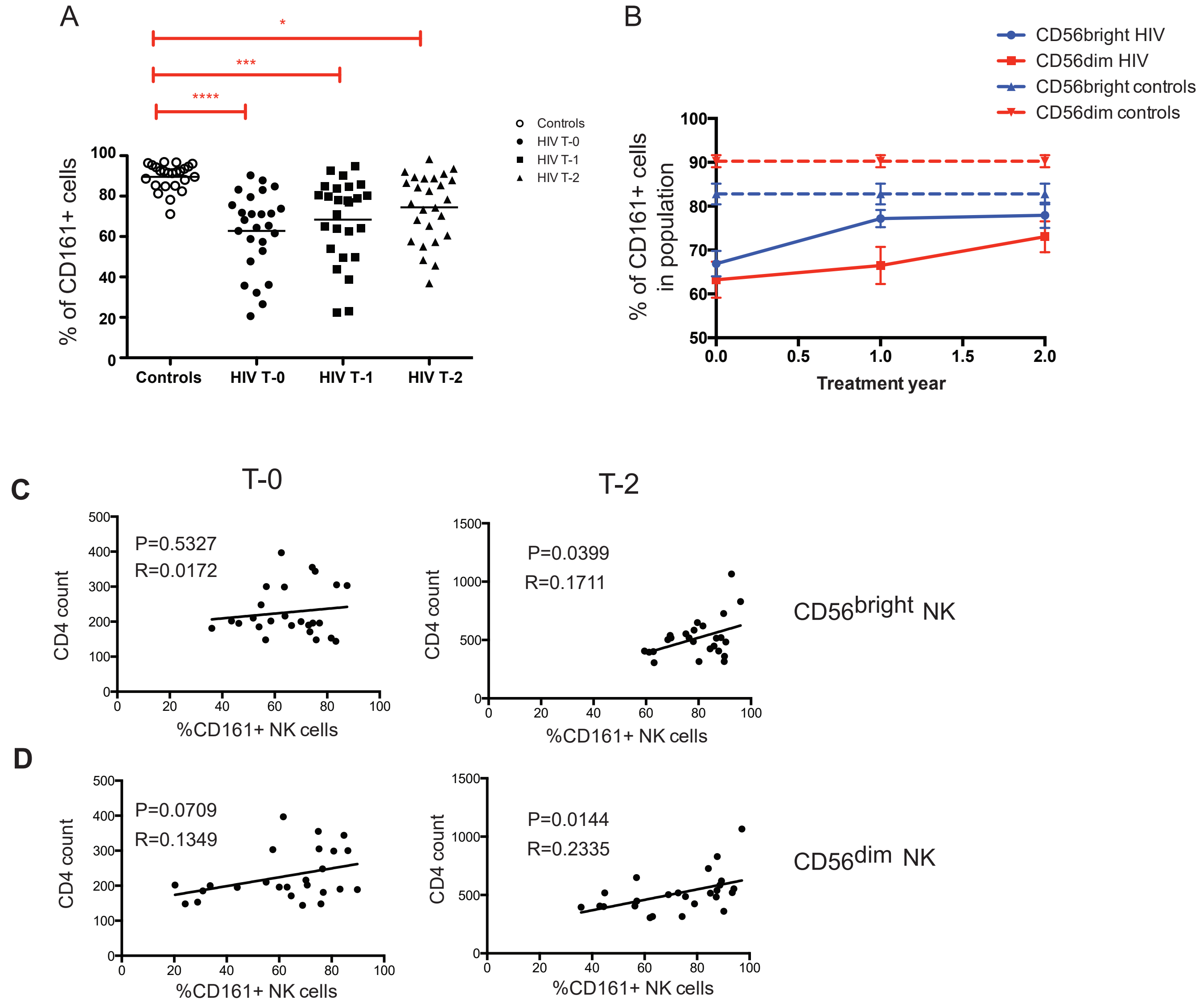

Supplementary Figure 7. Changes in CD161 expression on NK cells in HIV patients following anti-retroviral therapy. A-B) CD161 expression on NK cells in HIV patients, compared to healthy controls with unknown CMV serostatus. A) Expression of CD161 on total NK cells from healthy controls (n=24) or HIV patients (n=27) from the Swiss HIV cohort study were followed for two years of anti-retroviral therapy (ART), with samples taken prior to the start of the treatment (T-0), at one year (T-1), and two years (T-2) into treatment. ****p<0.0001, ***p<0.001, *p<0.05 by two-way ANOVA with Bonferroni's multiple comparisons test, compared to controls. B) The change in mean frequency of CD161+ NK cells within CD56dim NK cells (solid blue) and CD56bright NK cells (solid red) during two years of ART, compared to mean frequencies of CD161+ cells within CD56bright NK cells (dotted blue) and CD56dim NK cells (dotted red) in healthy controls (n=24). C-D) Correlation between CD4 counts and CD161 expression on C) CD56bright NK cells or D) CD56dim NK cells at T-0 and T-2. (N=27).
